# Distilling Heterogeneity of Mild Cognitive Impairment in the National Alzheimer Coordinating Center Database Using Latent Profile Analysis

**DOI:** 10.1001/jamanetworkopen.2020.0413

**Published:** 2020-03-06

**Authors:** Anna E. Blanken, Jung Yun Jang, Jean K. Ho, Emily C. Edmonds, S. Duke Han, Katherine J. Bangen, Daniel A. Nation

**Affiliations:** 1 Department of Psychology, University of Southern California, Los Angeles; 2 VA San Diego Healthcare System, San Diego, California; 3 Department of Psychiatry, University of California, San Diego; 4 Department of Family Medicine, University of Southern California, Los Angeles; 5 Department of Psychological Science, University of California, Irvine; 6 Institute for Memory Disorders and Neurological Impairments, University of California, Irvine

## Abstract

This cohort study uses latent profile analysis to examine the variation in mild cognitive impairment among participants in the National Alzheimer Coordinating Center (NACC) database.

## Introduction

Clinical trials aimed at improving mild cognitive impairment (MCI) and preventing dementia increasingly focus on biomarkers associated with risk, yet risk varies among individuals with MCI who have biomarker anomalies.^1^ Accurate cognitive phenotyping of MCI may improve prognostic accuracy. Recent studies have applied cluster analyses to neuropsychological data, identifying MCI cognitive endophenotypes.^2,3,4,5^ Observations across cohorts consistently identify cluster-derived mixed or dysnomic, dysexecutive, and amnestic groups, and a subgroup of individuals with MCI showing intact neuropsychological performance and attenuated dementia risk.^2,3,4,5^

Latent profile analysis (LPA) is a flexible, person-centered, and model-based clustering technique that enables probabilistic identification of neuropsychologically defined MCI subgroups. This cohort study aimed to extend prior work by applying LPA to a large, heterogeneous sample from the National Alzheimer Coordinating Center (NACC) database to delineate data-driven subgroups of persons with clinically diagnosed MCI and determine differential long-term risk for all-cause dementia.

## Methods

The NACC is a standardized, large-scale data set with clinical and neuropathological data from 39 Alzheimer Disease Centers. Each NACC site’s institutional review board reviewed and approved the study protocol before it could contribute data. Participants provided written informed consent according to Alzheimer Disease Centers Institutional Review Boards and completed semiannual physician and neurologic examination, medical history, and neuropsychological testing. Per NACC guidelines, an experienced clinician or consensus conference determined cognitive status. This cohort study followed the Strengthening the Reporting of Observational Studies in Epidemiology (STROBE) reporting guideline.

Baseline data were analyzed from 5155 participants with MCI and 12 490 participants who were cognitively unimpaired; participants were older than 60 years, had at least 1 follow-up visit between September 1, 2005, and March 15, 2019, and had results for at least 3 neuropsychological tests. Neuropsychological tests covering 3 cognitive domains were chosen, including memory (measured using the Logical Memory tests I and II), executive function (measured using the Trail Making Test parts A and B), and language (measured using the Animal Fluency Test and 30-item Boston Naming Test).

Analyses used Mplus version 8 (Muthén & Muthén) and SPSS version 24 (IBM Corp). We derived LPA clusters using raw neuropsychological scores. Participants who were cognitively unimpaired were included for comparison. Missing data were handled using missing-at-random maximum likelihood estimation. Akaike information criterion and bayesian information criterion were used to determine the optimal number of groups. The bootstrapped parametric likelihood ratio test compared models with 2 to 5 groups, based on previous literature.^2,4,5^ Cox regression and mean annual conversion rate (ACR) compared groups on rate of progression to dementia. Covariates included age, sex, self-reported race/ethnicity, and education. *P* values were 2-tailed, and statistical significance was set at .05. Data analyses were conducted from May 1, 2019, to August 21, 2019.

## Results

Data from 12 490 participants who were cognitively unimpaired (mean [SD] age, 73.2 [8.2] years; 5171 [41.2%] men; mean [SD] education, 15.3 [3.3] years) and 5155 participants with MCI (mean [SD] age, 75.8 [15.3] years; 2643 [51.3%] men; mean [SD] education, 15.3 [3.4] years) were analyzed. Fit criteria supported a 4-group solution. Our LPA revealed mixed dysexecutive and dysnomic (214 participants [4.2%]), dysexecutive (947 participants [18.4%]), and amnestic (2231 participants [43.3%]) MCI subgroups and a large, statistically determined neuropsychologically intact subgroup (1763 participants [34.2%]). The Table displays mean *z* scores and demographic characteristics. Notably, compared with other groups, the mixed MCI subgroup had the oldest mean (SD) age (amnestic: 76.1 [7.7] years; dysexecutive: 78.0 [8.3] years; neuropsychologically intact: 73.8 [7.8] years; cognitively unimpaired: 73.2 [8.2] years; mixed: 78.6 [8.5] years) and lowest mean (SD) years of education (amnestic: 15.5 [3.1] years; dysexecutive: 13.8 [3.7] years; neuropsychologically intact: 16.3 [2.8] years; cognitively unimpaired: 15.3 [3.3] years; mixed: 11.9 [4.6] years). Cox regression models (Figure) revealed significantly higher dementia risk among amnestic (hazard ratio [HR], 18.0 [95% CI, 15.2-20.0]; *P* < .001), dysexecutive (HR, 23.6 [95% CI, 20.7-26.9]; *P* < .001), and mixed (HR, 27.0 [95% CI, 21.9-33.4]; *P* < .001) MCI subgroups compared with the neuropsychologically intact subgroup (HR, 6.5 [95% CI, 5.7-7.4]; *P* < .001). Participants who were cognitively unimpaired demonstrated lower dementia risk (ACR, 1.6% [95% CI, 1.6%-1.6%]) than participants in the MCI subgroups (amnestic: ACR, 7.1% [95% CI, 6.9%-7.4%]; dysexecutive: ACR, 6.8% [95% CI, 6.3%-7.3%]; mixed: ACR, 6.7% [95% CI, 5.9%-7.8%]; neuropsychologically intact: ACR, 4.2% [95% CI, 4.1%-4.3%]; *P* < .001).

**Table.** Latent Profile Analysis Class Counts and Proportions Among Participants With MCI and Participants Who Were Cognitively Unimpaired

Characteristic	MCI (n = 5155)				Cognitively Unimpaired (n = 12 490)
	Amnestic (n = 2231)	Dysexecutive (n = 947)	Mixed (n = 214)	Neuropsychologically Intact (n = 1763)	
Men, No. (%)	1215 (54.5)	427 (45.1)	71 (33.2)	930 (52.8)	5171 (41.2)
Age, mean (SD), y	76.1 (7.7)	78.0 (8.3)	78.6 (8.5)	73.8 (7.8)	73.2 (8.2)
Education, mean (SD), y	15.5 (3.1)	13.8 (3.7)	11.9 (4.6)	16.3 (2.8)	15.3 (3.3)
Race/ethnicity, No. (%)^a^					
White	1914 (85.9)	645 (68.7)	107 (50.2)	1536 (87.3)	9955 (79.8)
Black	220 (9.9)	246 (26.2)	80 (37.6)	172 (9.8)	1953 (15.7)
American Indian or Alaska Native	8 (0.4)	12 (1.3)	3 (1.4)	4 (0.2)	109 (0.9)
Pacific Islander or Hawaiian Native	2 (0.1)	1 (0.1)	0	1 (0.1)	10 (0.1)
Asian	69 (3.1)	14 (1.5)	3 (1.4)	34 (1.9)	337 (2.7)
Other	14 (0.6)	21 (2.2)	20 (9.4)	13 (0.7)	109 (0.9)
Cognitive measure, *z* score, mean (SD)^b^					
Logical Memory I	−1.55 (0.81)	−0.99 (1.09)	−1.03 (1.06)	0.14 (0.83)	0.27 (1.11)
Logical Memory II	−1.74 (0.88)	−1.06 (1.06)	−1.13 (0.90)	0.09 (0.84)	0.30 (1.11)
Trails A	−0.21 (0.88)	−1.50 (1.21)	−5.98 (1.52)	−0.17 (0.89)	0.01 (1.24)
Trails B	−0.49 (0.87)	−3.65 (1.06)	−3.68 (1.19)	−0.33 (0.88)	−0.05 (1.25)
Animal Fluency	−0.97 (1.51)	−1.70 (1.80)	−2.91 (1.97)	−0.53 (1.06)	0.07 (1.17)
Boston Naming Test	−0.84 (0.88)	−1.01 (0.88)	−1.10 (0.82)	−0.39 (0.94)	−0.33 (1.21)

Abbreviation: MCI, mild cognitive impairment.

^a^ Includes data for 2227 participants in the amnestic MCI group, 939 participants in the dysexecutive MCI group, 213 participants in the mixed MCI group, 1760 participants in the neuropsychologically intact MCI group, and 12 473 participants in the cognitively unimpaired group.

^b^ Higher *z* score indicates superior performance, with 0 indicating average performance. A score less than or equal to 1.5 SDs below the mean is considered impaired.

**Figure.**
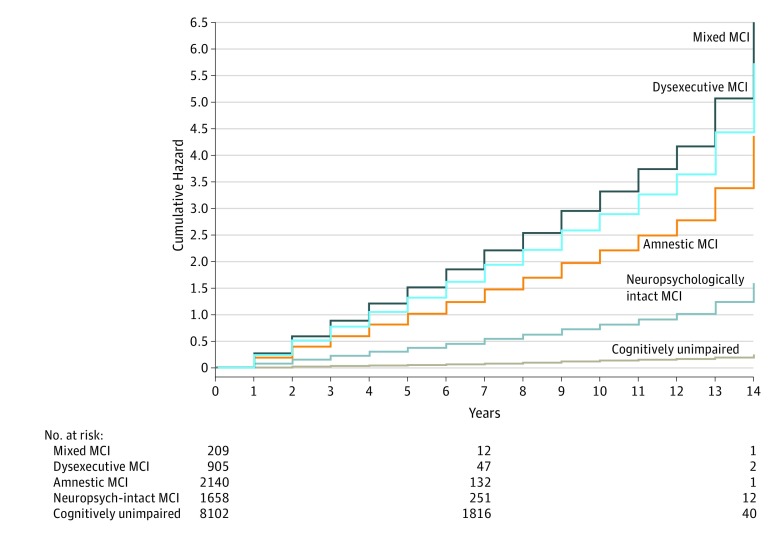
Relative Risks of Dementia Diagnosis Among Mild Cognitive Impairment (MCI) Subgroups and Cognitively Unimpaired Group

## Discussion

This cohort study found that the mixed and dysexecutive MCI subgroups were at the greatest risk for dementia, and the neuropsychologically intact MCI subgroup was at lowest risk despite showing a higher ACR than the cognitively unimpaired control group. The population MCI-to-dementia ACR is estimated to be between 5% and 10%,^6^ consistent with the ACRs we found for the mixed, dysexecutive, and amnestic MCI subgroups (6.7%-7.1%) but higher than the neuropsychologically intact MCI subgroup (4.2%). Notably, the neuropsychologically intact MCI subgroup constituted greater than one-third of the MCI sample, as observed in prior studies.^2,4^ Although neuropsychologically intact MCI may include persons with noncognitive (eg, neuropsychiatric) symptoms, many of these individuals may represent diagnostic errors contributing to MCI heterogeneity. Differences in demographic characteristics, symptom severity (eg, late vs early MCI), and symptom fluctuation may also contribute to differential dementia risk.^2,3,4,5^ Our findings extend prior work by demonstrating consistency of MCI phenotypes in a large, heterogeneous sample of participants with long-term follow-up.^2,3,4,5^ Our study has some limitations, such as that the cross-sectional examination of neuropsychological performance precludes us from making any conclusions about group differences in Alzheimer disease progression or phenotypic stability. Future research that includes biomarker data could improve characterization of dementia risk in MCI subgroups.^2^
